# Research trends in healthcare and hospital administration in Japan: Content analyses of article titles in the journal of the Japan society for healthcare administration

**DOI:** 10.3389/fpubh.2022.1050035

**Published:** 2022-12-14

**Authors:** Yasutoshi Moteki

**Affiliations:** School of Humanities and Social Sciences, Hiroshima University, Higashihiroshima, Hiroshima, Japan

**Keywords:** research trends, healthcare administration, hospitals, public medical insurance, content analysis, correspondence analysis, Japan

## Abstract

This study quantitatively analyzed healthcare administration studies in Japan using text mining, focusing on articles published during 1994–2021 in the *Journal of the Japan Society for Healthcare Administration* (prior to 2008, the journal was called *Hospital Administration*). Both the co-occurrence network and the correspondence analysis (these are extracted words that refer to the two systems) demonstrate two major changes: (1) the introduction of the long-term care insurance system, which was enacted in 1997 and came into effect in 2000, and (2) the introduction of the late-stage medical care system for the elderly in 2008, both of which had a significant impact on the Japanese public health and welfare system. *Co-occurrence network* and *correspondence analysis* were conducted to understand changes in research interests. The analysis used two time periods following a change in the journal's name in 2008. To readily comprehend changing research trends, 10-year segments were considered, resulting in three time periods. The research features and trends during the aforementioned periods were examined using correspondence analysis. Configuration figures derived from this analysis plotted *time transition* (first dimension) against certain *abstract/concrete situations* (second dimension). The extracted words displayed in the configuration maps at the axes' intersection were *patient, survey*, and *evaluation*. They revealed no distinctive features compared with other words and were commonly used in article titles within this journal during each period. The following results were obtained from the correspondence analysis: first, changes in the geriatric care system of public medical insurance and the introduction of the long-term care insurance system in 2000 were expressed in the characteristics of the extracted words; second, in the 14 years after the journal's name changed, published studies frequently referred to the roles of doctors, nurses, and other healthcare professionals. A chi-squared test on these extracted words and the period classification confirmed a statistically significant relationship between them.

## Introduction

The global spread of the novel coronavirus disease since 2019 has allowed researchers and practitioners in the healthcare field to challenge and reconsider the appropriateness of previous healthcare policies and hospital management practices. In Japan, the core functions of community healthcare have long been performed by public hospitals run by local governments. However, since the 1990's, economic constraints and a subsequent deterioration in national and local government finances have led to a reduction in the number of public hospitals and hospital beds, and a change in management methods from direct management to greater control by local independent administrative agencies. Amid the current coronavirus crisis, the importance of organizational flexibility and the need to improve the working conditions of medical personnel (including doctors, nurses, and clinical engineers who operate ventilators and extracorporeal membrane oxygenation), and ensure stable availability of personnel, have become critical issues. For instance, the workload of medical staff is increasing and their working environment is deteriorating. Hence, research is being conducted to find ways to optimize staff schedules (1). In this context, it is important to reflect on changes across research trends in healthcare administration regarding the efficient management of hospitals and hospital organizations to consider and implement future healthcare policy and management effectively. Concerning longer-term changes in the healthcare system and healthcare policy, since the 1980's, maturing of the Japanese economy and the declining birthrate and aging population have harmed the financial situation of national and local governments, thereby affecting the healthcare system. For instance, under these circumstances, the medical care system for older persons in Japan is being reviewed, with an increase in the co-payment ratio and financial separation from the general public medical insurance system.

This study examined the changing trends in healthcare administration research in Japan using content analysis and text-mining methods. Understanding changes in research trends can promote an understanding of the changing trends and representative cases regarding problems and how to address them in the field of healthcare administration. As research trends are somewhat related to actual institutional changes and trends in issues, quantifying these changes in the healthcare management field allows reconfirmation of the shifts in healthcare management systems that have been historically described, such as changes in universal health insurance systems. A content analysis method was proposed by Krippendorff (2) in the 1980's, focusing on the symbolism and meaning of documents and texts. The method was elaborated in subsequent studies (3–5). With the development of analytical technology and an increase in available software, the number of studies applying text-mining methods has increased. We employed a text-mining analysis, targeting article titles in the *Journal of the Japan Society for Healthcare Administration (Nihon Byoin Kanri Gakkai-shi)*. The journal was called *Byoin Kanri (Hospital Administration)* from 1964 to 2007. When the society's name changed in 2008, the journal's name also changed to its current one.

The volumes targeted for text mining ranged from volume 35 (1), published in 1994, to volume 58 (4), published in 2021. The volume numbers extend through the changes in the journal's name. The text-mining method is an enhanced version of content analysis, focusing on symbolic phenomena and semantic content in much social research. Krippendorff defines content analysis as “a research technique for making replicable and valid inferences from data to their context” (2 p. 21). With the development of computer technology, it has become possible to process greater amounts of content-related data than before, and this technique is often referred to as text mining. Researchers can also use the same open data generated to perform additional tests and reproduction studies, leading to more scientifically rigorous assessments to ensure verifiability.

In 1961, Japan's medical insurance system became universal, with all citizens covered by the public medical insurance system. The former Health Insurance Law, enacted in 1922, during the Taisho era (1912–1926), insured only manual laborers whose remuneration was below a certain amount in certain institutions with 10 or more employees. Later, in 1938, during the Showa era (1926–1989), a revised National Health Insurance Law was enacted. However, as enrollment was voluntary for both institutions and individuals, many citizens were not covered. Upon further revision of the National Health Insurance Law in 1958 and the implementation of universal health insurance in 1961, the public health insurance system has remained unchanged. The system differs greatly from the United States and other similar countries where most people subscribe to private medical insurance. However, two major changes have been introduced to the Japanese public insurance system, namely, the Health Care Law for the Elderly in 1983 and the Long-Term Care Insurance Law in 1997, which were in response to the rapid increase in the cost of medical care for older adults due to the aging of society in Japan.

This study analyzed changing trends in healthcare administration research in Japan using text-mining methods concerning article titles of the *Nihon Iryo Byoin Kanri Gakkai-shi (Journal of the Japan Society for Healthcare Administration)*. Prior studies have used bibliometrics in healthcare management and medicine to analyze research trends (6–10). Some of these studies (6–8) reviewed research trends related to new coronavirus infections *via* bibliometric analyses. Identifying research trends using bibliometric data is important in facilitating and positioning subsequent individual, original research. The text-mining analysis used in this paper is one of the research methods that uses bibliometric data.

The Japanese Ministry of Health, Labor, and Welfare states that Japan's public medical insurance system has the following four characteristics: one, all citizens are covered by public medical insurance; two, all citizens can freely choose medical institutions (free access); three, advanced medical care is provided at a low cost; four, public funds are invested in maintaining universal coverage, while the system is based on social insurance (11).

The Japanese public healthcare system has developed in response to ongoing economic challenges and the rapid aging of the population following an earlier period of high economic growth after World War II. According to definitions provided by the United Nations and the World Health Organization, a society with >7% of the population comprised of older persons is called an “*aging*” society; a society with a value of more than 14% is called an “*aged*” society, and that with a value of more than 21% is called a “*super-aged*” society (12). The aging rate in Japan has continuously risen, with Japan reaching the stage of an *aging society* in 1970, an *aged society* in 1994, and a *super-aged society* in 2007. First, in response to this evolving situation, the 1983 Health Care Law for the Elderly abolished the free medical care system for those aged 70 years and over (replacing the previous system of free medical care for older adults, which had been operating since 1973). Furthermore, medical insurance subscribers aged 75 years and over (65 years and over for those with disabilities above a certain level and certified under the Health Care Law for the Elderly) were classified separately from those insured under that age. The medical care system for older adults in these age groups is funded by public expenditure (derived from taxation) and contributions from various medical insurance systems, including public employee mutual aid societies (*Kyosai Kumiai*) and the health unions of general companies (*Kenko Hoken Kumiai*). The Long-Term Care Insurance Law, which passed in 1997 and came into effect in 2000, aimed to separate nursing care benefits from the medical care system, wherein medical insurance covered nursing care for older adults in convalescent beds in geriatric care, as symbolized by “social hospitalization.” The law established a public nursing care insurance system for those over 40 years with the basic local governments as the insurer, to clarify cost-sharing and benefits, and simultaneously secure financial resources for nursing care (13). The introduction of the long-term care insurance system in 2000, like the abolition of the free medical care system for older adults in the 1983 amendment to the Health Care Law for the Elderly, represented systemic reform to cope with the expansion of medical costs associated with the aging of society.

A further change was introduced concerning late-stage medical care for older adults in 2008. Older adults aged 75 and over are enrolled in an independent late-stage medical care system operated by a regional federation (of which the basic municipalities are members) and receive medical benefits (14). Finally, the 2015 Medical Insurance Reform Law resulted in the expansion of financial support for the national health insurance system, a gradual increase in the cost of meals at the time of hospitalization, and the introduction of a fixed fee for visits to large hospitals without a referral.

In the following paragraphs, a description of the selection process for target journal in applying text-mining analysis of research trends is presented. The following data on academic societies were derived from the *Gakkai Meikan* database of the Science Council of Japan, the Japan Science Support Foundation, and the Japan Science and Technology Agency (https://gakkai.jst.go.jp/gakkai/). The database was last updated on March 19, 2021, with results based on the fiscal year 2020 survey of academic societies. The following database searches were conducted in January 2022.

A textbook for practitioners in the field of medical management in Japan lists ten Japanese academic organizations related to hospital management: the Japanese Society of Medical Science, the Japanese Society of Internal Medicine, the Japanese Association of Surgeons, the Japanese Society for Healthcare and Hospital Administration, the Japanese Society of Nursing Science, the Japanese Society for Nursing Administration, the Japanese Society for Health Care Management, the Japanese Society for Clinical Pathology, the Japanese Society for Medical Work Assistance, and the National Association of Medical Affairs Research (15). Data concerning the first seven of these are available in the *Gakkai Meikan* database. The Japanese Society of Medical Science is an umbrella society for medical research, with the second, third, and fourth organizations listed being registered as subcommittees within it. Only the fourth and seventh organizations listed specifically target medical and hospital administration in general, including nursing administration. Table 1 summarizes the characteristics of these two societies based on the *Gakkai Meikan* database. As of July 2022, the *Gakkai Meik*an database is no longer in service. Therefore, the following information was valid as of January 2022, prior to the submission of this article. A list of academic organizations that meet certain criteria is available from the Science Council of Japan.

**Table 1:** Academic associations concerned with healthcare administration studies in Japan.

**Name of association**	**Establishment date**	**Name of the journal the association publishes**	**Number of members (individuals)**	**Number of members (legal entities)**	**Is the society affiliated with The Japanese association of medical sciences?**
Nihon Iryo Byoin Kanri Gakkai (Japan society for healthcare administration)	April 3, 1968	Until 2007, from 1964, the journal was named Byoin Kanri (Hospital Administration). From 2008 onwards, it is called the Journal of the Japan Society for Healthcare Administration	1,490 (regular member)	-	X
Nihon Iryo management Gakkai (Japan society for health care management)	1998 (Launch of the predecessor Critical Path Study Group)	*Journal of Japan Society for Health Care Management* (from 2000)	7,900	13	

Concerning the two academic societies presented in Table 1, this study chose to focus on the *Journal of the Japan Society for Healthcare Administration*, as the society has been in existence the longest and its related journal has the longest publishing history in the field. Therefore, it was likely to provide a clear picture of changing academic research trends. A sufficiently long publication history is needed to provide the necessary text data to track changes in research trends. In addition, the society's affiliation with The Japanese Association of Medical Sciences was stressed when selecting the target journal. The Japanese Association of Medical Sciences (https://jams.med.or.jp/index.html) is a comprehensive representative medical academic research network with 141 umbrella societies in Japan, which works in collaboration with the Japan Medical Association (https://www.med.or.jp/).

Content analysis or text-mining methods are increasingly used to analyze research trends in academic journals. Examples in various fields have been discussed previously (16). While Krippendorff (2) pioneered the content analysis method, Alcaide-Muñoz et al. (17) developed a quantitative method, specifically a science-mapping approach, to identify research trends in the field of local governments. Their research applied a bibliometric approach across e-government fields to visualize research trends and classify relevant studies by building a strategic and evolutionary map. The text-mining method used in this study also enabled the visualization of relevant characteristics and changes in research trends using correspondence analysis and co-occurrence network analysis. A quantitative analysis of articles published in the *Journal of Japan Society for Healthcare Administration* over 9 years, from January 2005 to December 2014, was previously conducted (18). This prior study considered the same journals as the current study. The previous study was in Japanese, with only the Abstract available in English. According to the Abstract, *nursing, systems*, and *management* were the three most frequently used words extracted in the analysis. Although the period covered was only 9 years, the study showed that from 2010 to 2014, the most frequently used terms extracted were related to the use of big data from Diagnosis Procedure Combination: DPC and receipts, home healthcare and home nursing, analysis-based management, and handling foreign patient care. The difference between their study and the current study is that the former also used the Abstracts of the target articles as data, whereas the current study only used the article titles and incorporated a longer analysis period. The current study used only the titles because they are more appropriate for understanding research trends, as titles are a straightforward description of the papers' themes. In addition, the long analysis period of 28 years enabled an accurate representation of changes in research trends. Furthermore, the publication of two quantitative analyses in the same journal enables the validity of the approaches to be verified.

Adunlin et al. (19) conducted a system analysis and bibliometric analysis of research trends in the medical field. Hao et al. (20) also used text-mining methods to conduct a bibliometric analysis of medical research trends. Khalil and Gotway Crawford (21) conducted a bibliometric analysis of behavioral risk factor surveillance studies in the United States. In the field of public administration, a field related to medical administration, Ni et al. (22) applied bibliometric methods to identify research trends in administrative studies published in the *Public Administration Review* concerning the United States. They selected 3,934 articles from the *Public Administration Review* and analyzed bibliometric data focusing on author attributes, showing the top 50 words most frequently used in the titles during three target periods. Many other bibliometric studies have also used the *Public Administration Review* as an object of analysis (23–26).

## Materials and methods

### Collection of materials

This study quantitatively examined research trends in hospital administration studies after the 1980's in Japan using article titles published in the *Journal of the Japan Society for Healthcare Administration*, ranging from volume 35 (1), in 1994, to volume 58 (4), in 2021. This is a total of 28 years, including 14 years each before and after the change in journal name in 2008 [the new name applied from volume 45 (1) onwards].

The article titles from the *Journal of the Japan Society for Healthcare Administration* were gathered to determine post-war research trends in hospital administration studies in Japan. The search results were saved as Excel spreadsheet files in tab-separated values format (implemented on November 28, 2021). As the CiNii Articles database only contains volumes from 1995, articles from four issues of volume 35 in 1994 were inputted by the author and added to the dataset based on the tables of contents of originally published journals. The table of contents information provided by the National Diet Library for the journal was used to check the accuracy of the journal titles. The data from volume 35 to volume 58 were consolidated into one dataset file in Excel spreadsheet format. The dataset file was translated from Japanese to English using the Google Translate application. The dataset used machine translation to facilitate replication and follow-up research by third-party researchers. The author read the entire translated text, confirming the appropriateness of the machine translation.

The author then made the following modifications to the dataset file: first, titles, such as “Preface,” “Editor's Postscript,” “Special Remarks,” “Special Lectures,” “Annual Meeting,” “Inauguration of the President,” and “General Contents,” and “book reviews” were omitted; second, non-research articles with blank author names that usually comprised administrative communication articles concerning the association and annual conferences were also omitted. The final dataset comprised 692 articles from the journal. The author used decade periods to capture changes in research trends. The resulting three-period classification was used in the analysis of the extracted words.

To analyze the text data of the articles in the journal, KH Coder (version 3.Beta.04a), produced by Dr. Koichi Higuchi of Ritsumeikan University, was employed because it is free to use and has advanced visualization features for conducting correspondence analysis. Higuchi (27) provides details concerning the software and an application example for real text materials. KH Coder offers a choice of several methods for processing English extracted words, and the Stanford POS Tagger was chosen for morphological analysis of sentences (this option is the default for KH Coder, but other options include FreeLing, and Snowball). POS Tagger (Part-Of-Speech Tagger) is a generic term for software that focuses on parts of speech, such as nouns and adjectives, to segment sentences. Stanford POS Tagger is one POS Tagger software package, officially named the Stanford Log-linear Part-Of-Speech Tagger (https://nlp.stanford.edu/software/tagger.shtml). The statistical analysis used tests such as the χ2 test. The significance level was defined as 5%.

## Results

The dataset was loaded into KH Coder for the text analysis, and the extracted words were pre-processed using Stanford POS Tagger. The author conducted correspondence and co-occurrence network analyses to graphically depict the relationships between the extracted words. By drawing configuration maps as revealed in the correspondence analysis with 10-year periods as external variables, the changes in and characteristics of each period in terms of healthcare administration studies in Japan were visualized.

### Co-occurrence network analysis

**Figure 1:**
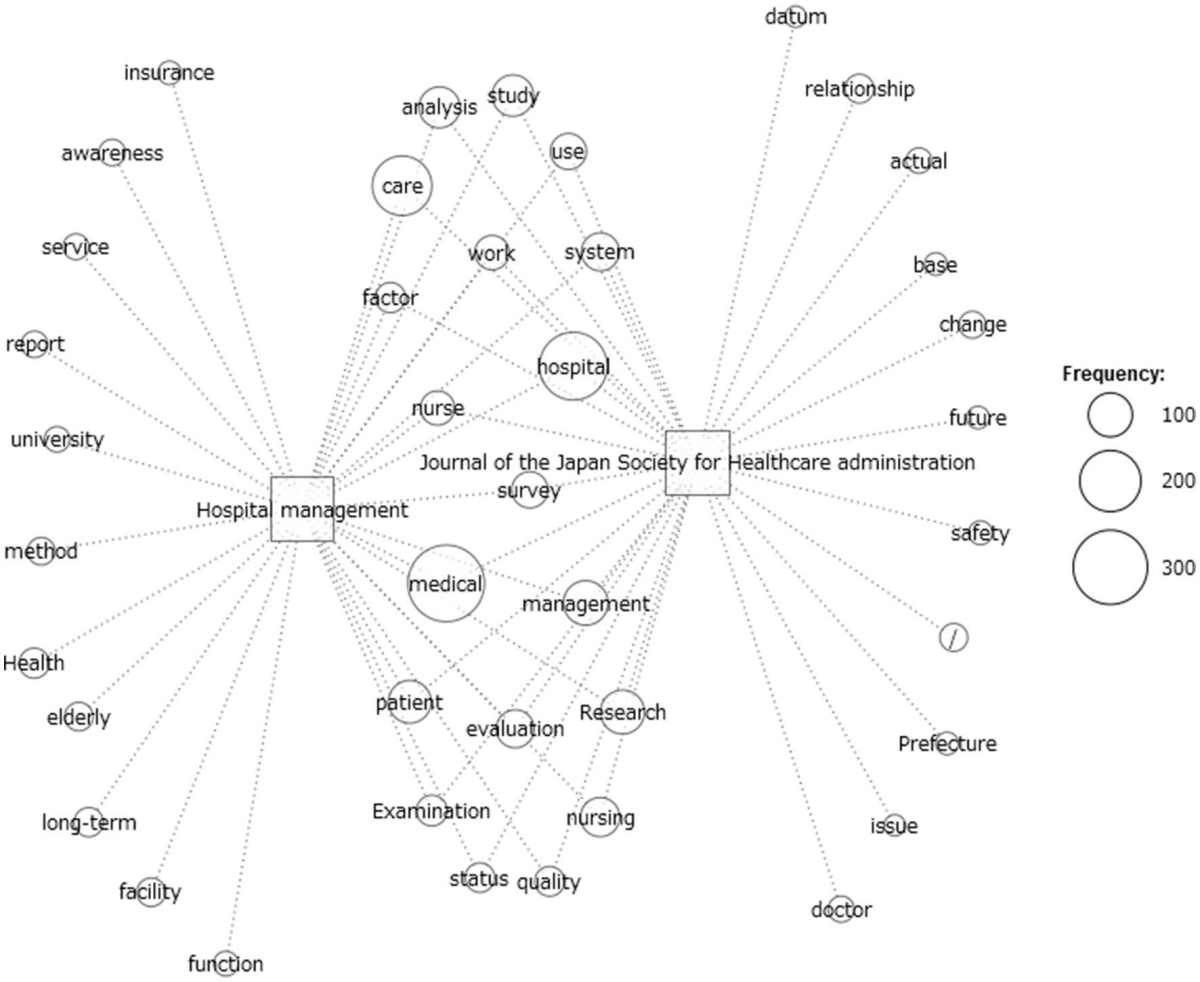
Co-occurrence network analysis of the extracted words.

The co-occurrence network analysis with 60 filtering edges is shown in Figure 1. A co-occurrence network analysis was performed to understand the change in focus before and after the change in the journal name from January 2008. The year 2008 saw the introduction of the late-stage medical care system for older adults, a trend aimed toward separating the elderly care component within public medical insurance, and a greater injection of public funds. Using the two periods before and after the change in the public medical insurance system in 2008 was considered likely to facilitate a good understanding of differing research trends during the pre-and post-2008 periods. This study used the Jaccard index to determine the selection criteria (28); the Jaccard index is the default option for KH Coder. A detailed mathematical explanation of the Jaccard index is provided in the aforementioned reference.

We found an extensive use of the extracted words *prefecture, relationship, change, future, safety*, and *doctor*, among others, for the period 2008 onwards (see the right side of Figure 1). The year 2008 marked the introduction of the late-stage medical care system for the elderly in Japan. Under this system, the medical care system for the elderly, which covers about 16.1 million people aged 75 and over, is distinguished from other age groups, and a 4:1:1 ratio was set between the national, prefectural, and municipal governments for. In addition, as for the portion of the cost borne by public health insurance, the financial burden from the younger age groups to the later elderly generation was set at about 40%. The word *prefecture* refers to the prefectures that now account for about 16.7% of the public funding that makes up half of the late-stage medical care system for the elderly (about 8.3% of the total). This major change in the public health care system can be noticed in the words *change* and *future*.

On the left side of Figure 1, the words *insurance, elderly*, and *long-term* were extensively used in the journal prior to January 2008 (from 1994). These words refer to the introduction of a new healthcare system for older adults in 1997, which was actively discussed at conferences and in published papers. In other words, this major change lies in the passing of the Long-Term Care Insurance Law in 1997 and its implementation in 2000. The enactment of the Long-Term Care Insurance Law was a major change to Japan's health and welfare system, primarily by separating the provision of long-term care services to the elderly from the medical insurance system and transferring them to public long-term care insurance, and changing the focus of welfare services from government directive measures to citizens' contracts with private providers. The extracted words *care, medical, hospital, evaluation, management, patient, nurse, examination, system*, and *quality* were commonly featured in studies of healthcare administration in Japan throughout the two periods, as shown in their predominance at the center of Figure 1.

### Correspondence analysis

This section examines the relationship between the extracted words using the correspondence analysis method, which facilitates a graphical display of categorical data. A 10-year period was set as the external variable. Thus, three 10-year periods were used to display relevant characteristics and changes in healthcare administration studies over time in Japan. The final 2 years, 2020–2021, were included in the preceding period, 2010–2019, because the period 2020–2021 was too short. To facilitate verification by other researchers, the author did not limit the target focus in this study, as narrowing the target parts of speech or excluding certain extracted words can inhibit the process of conducting the correspondence analysis. For this reason, extracted words unrelated to the research theme, such as the slash symbol, are shown in the following analysis results.

**Figure 2:**
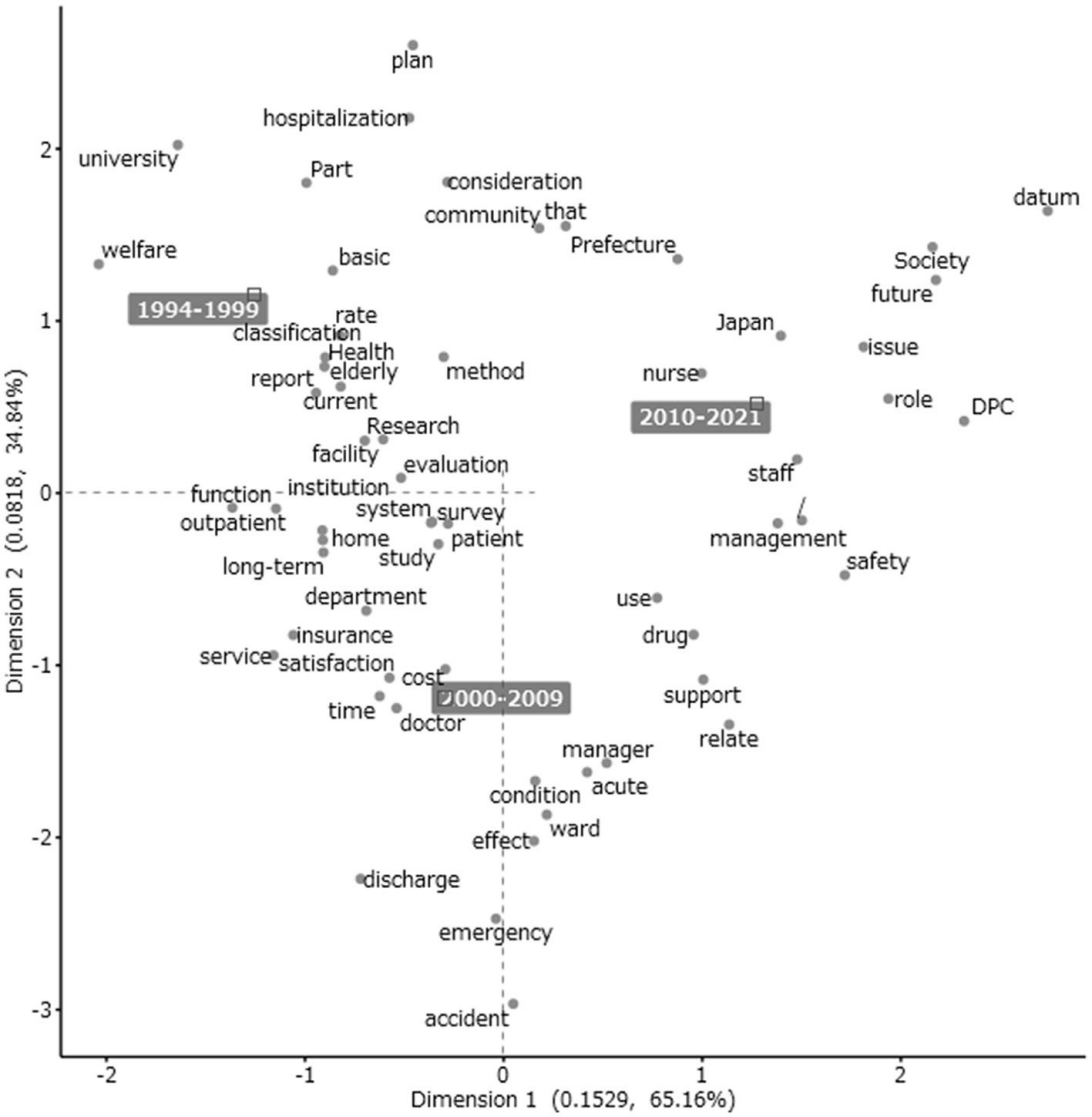
Correspondence analysis (dimension 1 [time] and dimension 2 [abstract/concrete situations]).

The results of the correspondence analysis based on the three periods are presented in Figure 2. Extracted words strongly associated with each time period category are indicated near each period display. The words *welfare, classification, hospitalization*, and *university* appeared in the upper left quadrant of Figure 2, near the 1994–1999 display. These extracted words are related to the content of studies conducted between 1994 and 1999. The words *nurse, Japan, staff, management*, and *safety* appear on the right side of the configuration map, near the 2010–2021 display. In Figure 2, the chronological indication with a black background presents the variables of the periods (white on black) of 1994–1999, 2000–2009, and 2010–2021 in a left-to-right time transition pattern. The extracted words located near the origin where the first and second dimensions intersect, such as *survey, patient*, and *evaluation*, had no distinctive features compared to the other words. These words were often used regardless of the period and are characteristic of the diachronic research trends in this field. The most influential axis from left to right was the time dimension in the configuration map (the contribution ratio was 65.16%).

The words *doctor, manager, cost, acute, insurance, service, satisfaction*, and *condition* appear in the lower part of Figure 2, near the 2000–2009 display. The word *acute* forms part of the expression “severe acute phase” and is used in relation to the type of hospital involved—the English title of Yoshida et al. (29) includes the phrase “severe acute pancreatitis.” The severe acute phase is called *Kyuuseiki* in Japanese and is used in combination with the chronic phase, known as *Manseiki* in Japanese. Differences in the values of dimension 2 are expressed as differences in positioning between the top and bottom of Figure 2. The words at the far end represent the most characteristic of that axis, so *accident* and *emergency* are one pole and *plan* the other. The word *plan* is located at the highest position, and extracted words related to medical policy, such as *university* and *hospitalization*, are also found in the upper part of Figure 2. In the lower part of Figure 2, the words *accidents, emergencies*, and *discharges* appear, indicating types of policy and specific situations in hospitals. Dimension 2 can be interpreted as an axis representing certain abstract/concrete situations. Although the interpretation of dimensions is subjective and alternative interpretations are possible, such interpretation is often used in correspondence analysis to facilitate subsequent scholarly discussion.

The extracted words located in the earlier period had no distinctive features compared to the other words. The extracted words displayed in the configuration map at the intersection of the two axes, around the center of Figure 2, are *patient, survey*, and *evaluation*, which were commonly used words in the titles of articles published in this journal, regardless of the period.

The words *doctors, staff, nurses, nursing, managers, roles*, and *relationships*, which relate to the occupations, roles, and interrelationships of medical professionals, were tagged to include them together in subsequent analyses. Table 2 shows a cross-tabulation in relation to whether the title of each article included or did not include these tagged extracted words and the two periods (pre- and post-2008) by journal name. A χ2 test conducted with the data presented in this table revealed a chi-squared value of 3.87, indicating a statistically significant association between the two variables at the 5% level.

**Table 2:** Target words included or not included pre- and post-2008 in the journal.

	**Included (1)**	**Not included (0)**	**Total**
Journal of the Japanese society on hospital administration (pre-2008)	68	314	382
	17.8%	82.2%	100.0%
Journal of the Japan Society for Healthcare Administration (2008 and post-2008)	74	236	310
	23.9%	76.1%	100.0%
Overall	142	550	692
	20.5%	79.5%	100.0%

Target words: Doctors, staff, nurses, nursing, managers, roles, and relationships.

## Discussion

This study quantitatively analyzed research trends using data mining in healthcare administration studies in Japan, surveying article titles spanning 28 years taken from the *Journal of the Japan Society for Healthcare Administration*. Most significantly, it was found that through the co-occurrence network analysis and correspondence analysis, we were able to find two major changes in the Japanese health and welfare system among the extracted words: first, the introduction of the long-term care insurance system in 2000 and second, the late-stage medical care system for the elderly introduced in 2008. In the correspondence analysis, with three 10-year periods set as an external variable, the first dimension of the configuration figures was identified as an axis of time transition, and the second dimension concerned abstract/concrete situations regarding the placement of extracted words in the configuration map. The extracted words located in the earlier period had no distinctive features compared to the other words. The extracted words displayed in the configuration map at the intersection of the two axes, around the center of Figure 2, were *patient, survey*, and *evaluation*, which were commonly used in the titles of articles published in this journal, regardless of the period.

This study is novel in that it analyzed research trends in healthcare administration and hospital administration management in Japan using quantitative methods and clarified relevant diachronic characteristics and changes over time. Changes in practice within the Japanese healthcare administration were reflected in the prevalent research trends in the target journal. Through co-occurrence network analysis and correspondence analysis, changes in research trends in academic societies that corresponded to changes in Japan's public medical insurance system could be identified.

### Limitations

One limitation of this study is that it covered only a certain period in a single journal. Adding other journals to the analysis and comparing individual results can be addressed in future studies. A similar analysis of other journals would help validate the results of this study's analyses and simultaneously clarify potentially differing research trends among the various academic societies and associations. The author intends to conduct such a comparison in future studies through an analysis of the *Journal of the Japan Health Care Management*, in order to verify the current findings. In addition, quantitative analyses have certain limitations. A text-mining analysis of journal article titles focuses on the frequency of extracted words in the article titles. Therefore, it is possible that studies with infrequently used words that have a decisive impact could be overlooked. In addition, the same phenomenon may be described using different words. This problem needs to be addressed by tagging or grouping extracted words. In addition, this text mining analysis focused on nouns, which may have led to some elements being left out. There are certainly limitations to the part-of-speech analysis based on morphological analysis, for which artificial intelligence methods, which are currently on the rise, will eventually be developed so as to get closer to the actual meaning. The author would further like to study new methods and apply them to the field of this research.

### Future research agendas

This was a meta-analytical study that addressed research trends rather than individual empirical studies. Reviewing past changes in academic research trends and accumulating individual empirical studies in the academic community is important. Such efforts could result in better progress in healthcare administration studies. As mentioned above, the author would like to continue analyzing research trends in Japanese healthcare administration using quantitative text mining methods or meta-analysis techniques.

## Data availability statement

The raw data supporting the conclusions of this article will be made available by the authors, without undue reservation.

## Author contributions

The author confirms being the sole contributor of this work and has approved it for publication.
